# Improved Recovery of Complete Spinal Cord Transection by a Plasma-Modified Fibrillar Scaffold

**DOI:** 10.3390/polym16081133

**Published:** 2024-04-18

**Authors:** Diana Osorio-Londoño, Yessica Heras-Romero, Luis B. Tovar-y-Romo, Roberto Olayo-González, Axayácatl Morales-Guadarrama

**Affiliations:** 1 Electrical Engineering Department, Universidad Autónoma Metropolitana, Mexico City 09340, Mexico; dmol@xanum.uam.mx; 2 Experimental Analysis of Behavior Department, Faculty of Psychology, Universidad Nacional Autónoma de México, Mexico City 04510, Mexico; heras@ifc.unam.mx; 3 Department of Molecular Neuropathology, Instituto de Fisiología Celular, Universidad Nacional Autónoma de México, Mexico City 04510, Mexico; ltovar@ifc.unam.mx; 4 Physics Department, Universidad Autónoma Metropolitana, Mexico City 09340, Mexico; 5 Medical Imaging and Instrumentation Research National Center, Universidad Autónoma Metropolitana, Mexico City 09340, Mexico

**Keywords:** spinal cord transection, electrospun scaffolds, plasma synthesis, MRI

## Abstract

Complete spinal cord injury causes an irreversible disruption in the central nervous system, leading to motor, sensory, and autonomic function loss, and a secondary injury that constitutes a physical barrier preventing tissue repair. Tissue engineering scaffolds are presented as a permissive platform for cell migration and the reconnection of spared tissue. Iodine-doped plasma pyrrole polymer (pPPy-I), a neuroprotective material, was applied to polylactic acid (PLA) fibers and implanted in a rat complete spinal cord transection injury model to evaluate whether the resulting composite implants provided structural and functional recovery, using magnetic resonance (MR) imaging, diffusion tensor imaging and tractography, magnetic resonance spectroscopy, locomotion analysis, histology, and immunofluorescence. In vivo, MR studies evidenced a tissue response to the implant, demonstrating that the fibrillar composite scaffold moderated the structural effects of secondary damage by providing mechanical stability to the lesion core, tissue reconstruction, and significant motor recovery. Histologic analyses demonstrated that the composite scaffold provided a permissive environment for cell attachment and neural tissue guidance over the fibers, reducing cyst formation. These results supply evidence that pPPy-I enhanced the properties of PLA fibrillar scaffolds as a promising treatment for spinal cord injury recovery.

## 1. Introduction

Complete spinal cord injury (SCI) disrupts the neural tissue irreversibly, affecting motor, sensory, and autonomic functions. The primary injury produces a chain of events at the injury site called secondary damage, aggravating the injury outcome. This secondary injury induces the production of free radicals, edema, ischemia, hypoxia, excitotoxicity, and the formation of a glial scar at chronic stages, constituting a microenvironment that causes the disconnection of neural circuits and failure of neural tissue regeneration [1,2]. Despite extensive research on spinal cord injury therapies for the recovery of motor, sensory, and autonomic functions, currently, there are no effective treatments to completely restore tissue structure and function [3,4].

Current treatments commence prehospitally, with on-site management, cardiovascular function monitoring, immobilization, and rapid transferring to a center capable of providing intensive care, accurate diagnosis using techniques such as computer tomography (CT) and magnetic resonance (MR) imaging, and surgical interventions such as decompression. As a result, aggravation of the primary injury can be avoided in most cases [5]. The use of pharmacological treatments such as methylprednisolone is controversial, due to the associated adverse effects and modest improvements [6]. Many therapeutic strategies have been proposed, including neuroprotection, structural repair, and neural stimulation strategies [5]. Neurorehabilitation is also recommended as early as possible since it enhances neuroplasticity, neurotrophic factors secretion and promotes regeneration. However, in the case of complete SCI, no spared pathways can be stimulated, and therefore other strategies such as restorative scaffolds have been proposed. 

Due to the inherent complications of SCI and its heterogeneity, despite the plethora of therapies proposed [7], limited functional recovery (motor, sensory, and autonomic), allodynia and chronic neuropathic pain management, bladder and bowel dysfunctions, secondary complications (respiratory conditions, cardiovascular disease, metabolic disorders, and muscular atrophies), psychological problems such as depression and anxiety, and the inequality of access to health among individuals of different populations [8] remain as unmet medical problems in the field of SCI [9,10,11].

The natural progress of the secondary injury results in the formation of a fluid-filled cavity surrounded by fibrotic scarring, collapsing the neuronal regenerative impulses [2,12]. It has been proposed that providing a biocompatible platform for spared neural tissue to attach and reconnect across the injury [13,14,15], taking advantage of the frequently necessary surgical intervention after SCI [16,17], could be a potential treatment strategy for acute injury. Chronic treatment for SCI has also been proposed using the scaffold implant strategy after surgically removing the fibrotic scar tissue [18,19,20,21] to activate regenerative processes supported by permissive scaffolds. 

From a tissue engineering perspective, strategies including biocompatible materials that provide the basis of secondary injury modulation and act as a bridging platform across the injury epicenter are presented as attractive potential treatments since they provide support for endogenous as well as transplanted cellular repairing agents [4,20,22,23,24]. 

Biomaterial scaffolds have been extensively applied as a promising strategy for SCI since, fundamentally, scaffolds provide structural support for cell attachment, migration, tissue growth, repair, and regeneration [5,24,25]. Nevertheless, considering the complex structure of the central nervous system and the physiopathology of SCI [26], the clinical use of biomaterial scaffolds for spinal cord recovery has only been applied in a handful of patients [13,15,19,27,28]. The optimal design of biomaterials for SCI treatment constitutes a current research problem. 

The plasma-synthesized pyrrole polymer doped with iodine (pPPy-I) is a biocompatible material that has been studied as an implant, in the form of particles in suspension in contusion spinal cord injury models [29,30,31,32], and as tablets in transection models [33,34,35] in rat [29,30,31,32,33,34,35] and non-human primate [14] models. These studies demonstrated that pPPy-I supports neurons and growth cones at the injury site, myelination, neuroprotection, cell adhesion, survival, and differentiation. PPPy-I also moderates inflammatory responses and the effects of secondary injuries, such as cysts and glial scar formation, and produces motor recovery [29]. However, multiple studies have highlighted the importance of integrating various cues, namely, topographical, chemical, and mechanical guidance for functional recovery strategies for SCI [25,26,36]. 

Scaffolds for neural tissue engineering have been prepared by techniques such as 3D printing, electrospinning, decellularized tissue, and hydrogel design [25,26], with the purpose of obtaining a substrate that mimics the extracellular matrix and promotes recovery. Mechanical compliance, bioactivity, porosity, the surface-to-volume ratio, topography, and architectural properties determine the tissue response, and in the specific case of SCI, modifying the cytotoxic milieu associated with injury physiopathology is one of the most relevant attributes of optimal scaffolds to provide the adequate cues to drive repair and regeneration processes. 

Polylactic acid (PLA) is a biocompatible material that affords attractive physicochemical, mechanical, and rheological properties [37,38,39,40]. Due to its versatile processability, PLA has been used in many applications [25,41,42], such as devices fabricated using different techniques, such as 3D printing or electrospinning [40]. In particular, electrospinning produces scaffolds that can mimic the extracellular matrix, both its architectural and mechanical properties, by adequately configuring the process parameters [26,43,44,45]. Finetuning the scaffolds’ properties such as porosity, the surface-to-volume ratio, topography, bioactivity, and mechanical compliance constitutes a continuously updated research field [45]. Nevertheless, due to the limited hydrophilicity of PLA, surface modification is usually applied in tissue engineering applications [25,44,46]. 

In this work, a PLA scaffold was coated with pPPy-I (PLA + pPPy-I) and implanted as a therapeutic strategy in a complete spinal cord injury transection model in adult rats in order to evaluate if adding a structural component to pPPy-I provided enhanced structural reconstruction and functional recovery. The model was evaluated by MR imaging, a non-invasive in vivo technique that enables the investigation of the nervous tissue structure. Imaging techniques, such as diffusion tensor imaging (DTI) and MR spectra acquisition (MRS), were used to further investigate tissue constitution in response to injury and implant, increasing imaging sensitivity and leading to more accurate diagnostics [47]. Finally, histology and immunofluorescence studies were performed. MAP2, a neuronal marker found in the dendrites of mature neurons [48], and DAPI, which is associated with the nucleus of cells, were used for immunofluorescence studies. These markers were applied to assess neural tissue growth through the fibers of the scaffolds.

## 2. Materials and Methods

### 2.1. Materials

Polylactic acid (PLA Ingeo 3251D, Minnetonka, MN, USA) amorphous biopolymer, chloroform (J. T. Baker, Avantor Performance Materials, Inc., Radnor, PA, USA), dimethylformamide (J. T. Baker, Avantor Performance Materials, Inc., Radnor, PA, USA), Pyrrole, and iodine (Sigma-Aldrich, St. Louis, MO, USA,) were used to prepare the scaffolds. Xylazine (PiSA Agropecuaria S.A. de C.V.), pentobarbital (PiSA), and Zoletil (Virbac, México) were used as anesthetics. Adult female Wistar rats (230–290 g) from the Metropolitan Autonomous University (Iztapalapa) Bioterium were used for this study. Buprenorphine (PiSA) and enrofloxacin (Enroxil, Senosiain) were used for analgesic and antibiotic therapy, respectively. Sodium chloride (NaCl), paraformaldehyde (PFA), phosphate buffer (PB) and Tris-Buffered Saline (TBS) components, bovine serum albumin (BSA), and Triton X-100 were purchased from Sigma-Aldrich. For immunofluorescence, Anti-MAP2 (Invitrogen, Waltham, MA, USA, PA5-17646) and Alexa Fluor 546 anti-rabbit secondary antibody (Thermo Fisher Scientific, Waltham, MA, USA, A11035) were used.

### 2.2. Scaffold Design

PLA fibrillar scaffolds were prepared using electrospinning and coated with pPPy-I, as previously reported [44]. A 15% *v*/*v* PLA solution was pumped through a nozzle connected to a 20 kV high-voltage electric field source. The fibers were collected on a grounded plate, generating a porous scaffold, which was folded to form 3 × 3 × 1 mm rectangular-shaped constructs, as previously reported [49]. The constructs were placed inside a glass reactor between two electrodes connected to a radiofrequency generator. The pyrrole (Py) monomer and iodine (I) dopant were introduced through valves to the reactor chamber, and synthesis was conducted for 30 min at a 1 Torr pressure. A crosslinked, branched polymer forms the resulting coating (pPPy-I) without any remnant oxidant compounds, presenting semiconducting properties in the physiological environment [50] and functional groups with affinity to the cell membrane and extracellular matrix-binding integrins [51].

### 2.3. Animals

Adult female Wistar rats weighing 230–290 g were used. Rats were housed in groups of 4–5 individuals per cage, inside a temperature-controlled room, under a 12-h light/12-h dark inverted cycle (light hours during nighttime) [52,53,54]. Food and water were provided ad libitum. Sanitary bedding was changed regularly to ensure the rats were dry.

All the experiments complied with the official Mexican specifications NOM-062-ZOO-1999, the National Research Council Guide for the Care and Use of Laboratory Animals [55], ICLAS, and ARRIVE guidelines. The animal study protocol was approved by the Institutional Ethics Commission of the Health and Biological Sciences Division of the Metropolitan Autonomous University (protocol code CECBS22-05 approved on 18 November 2022). 

### 2.4. Study Design

The complete spinal cord transection (CSCT) addressed the spinal cord injury study to abolish remaining nerve tracts that could produce plasticity processes and cloud the therapeutic effects of the scaffold treatment. 

Male rats were not considered to avoid complications due to hind-body dragging and neurogenic bladders. Exclusion criteria were animals with motor recovery within the first five days after injury, evidence of remaining nerve tracts by magnetic resonance (MR) imaging, and muscular atrophy due to hindlimbs biting. MR studies that presented movement artifacts and MR spectra with low signals, which could not be adjusted to selected peaks, were not considered. 

We used a total of 68 female adult Wistar rats. The animals were randomly assigned to one of four experimental groups, namely, intact animals without injury (n = 7), complete transection only (Control, n = 19), PLA (n = 20), and PLA + pPPy-I (n = 22) implants, and studied for eight weeks, which is the typical period of rat recovery [56]. Magnetic resonance, histology, and immunofluorescence studies were run in independent studies. 

### 2.5. Spinal Cord Transection

The animals were anesthetized intramuscularly with Xylazine (10 mg/kg) and Zoletil (10 mg/kg) [57,58]. Once deep anesthesia was confirmed by toe pinching, the back of the animal was shaved, and povidone-iodine was applied to the incision area. A laminectomy was performed on the 9th thoracic vertebra using sterile materials to expose the meninges. An approximately 3mm incision was made in the meninges to expose the spinal cord. The spinal cord was transected using micro scissors, and the complete transection model was verified by inserting a fine tip into the entire cut area [34,59]. The implant was inserted between the spinal cord stumps, and the control group received no implant. Then, a fabricated patch made of a 5 × 3 mm single mesh of PLA + pPPy-I was placed over the incision of the meninges of all the animals. Muscle and skin were layered closed using absorbable polyglycolic acid (PGA) and nylon sutures, respectively. The animals were left to recover next to a heat source until fully awake. Analgesic (0.05 mg/kg Buprenorphine) and antibiotic (5 mg/kg enrofloxacin) therapy was conducted for three days, and the bladder was manually voided twice daily [60]. 

The animals were monitored daily, taking care of any signs of excessive pain or disease [61]. Antibiotics, analgesics, wound cleaning, or euthanasia were applied when necessary to ensure the animals’ comfort. The mortality rate due to CSCT was 24%. Body weight was also monitored as a measure of general animal health, and the loss of >20% of the body weight criterion was considered the humane endpoint [60,62].

### 2.6. Magnetic Resonance Imaging (MRI) Acquisition

MRI studies were performed during the first, fourth, and eighth weeks of treatment in a 3T Philips Achieva Medical System coupled to a 16-channel neurovascular coil. T1-weighted (T1W) and T2-weighted (T2W) sequences were acquired for anatomical analysis. T1W 3D was acquired using a Turbo field echo (TFE) Gradient Recalled sequence with TE = 4.6 ms (echo time), TR = 9.9 ms (repetition time), 384 × 384 matrix, 0.8 × 1.08 × 0.8 mm voxel size, field of view (FOV) of 246 × 246 mm, 140 slices 0.8 mm thick, −0.4 Gap, and a number of signal averages (NSA) of 7. The T2W Spin Echo sequence was acquired sagittally, TE = 80 ms (echo time), TR = 3000 ms (repetition time), 512 × 512 matrix, FOV = 230 × 230 mm, 0.6 × 0.62 × 2 mm voxel size, 24 slices 2 mm thick, no gap, NSA 4.

A Diffusion tensor imaging (DTI) scheme was used to acquire the diffusion images transversally, at the injury region, in 33 diffusion sampling directions, with a b-value of 800 s/mm^2^. TE = 84 ms (echo time), TR = 2521 ms (repetition time), 1.51 × 1.54 × 1.5 mm voxel size, FOV = 224 × 224 mm, 160 × 160 matrix, 20 slices, no gap, NSA 9. In-plane resolution and slice thickness were 1.4 mm and 1.5 mm, respectively. DSI Studio software (Dec 19, 2019 build, https://dsi-studio.labsolver.org/, accessed on 15 April 2024) was used to process the DTI data. The diffusion tensor was calculated, and a deterministic fiber tracking algorithm [63] was used. A region of interest (ROI) was placed at the spinal cord with a volume size of 327 ± 64 mm^3^. A seeding region was placed in the whole ROI. The anisotropy threshold was 0.2. The angular threshold was 40 degrees. The step size was 0.2 mm. The fiber trajectories were smoothed by averaging the propagation direction with 50% of the previous direction. Tracts with lengths between 1 and 300 mm were kept. Topology-informed pruning [64] was applied to the tractography with 2 iteration(s) to remove false connections.

Magnetic resonance (MR) spectra were obtained at the injury site with a PRESS (point-resolved spectroscopy) sequence with water suppression, TE = 144 ms (echo time), TR = 2000 ms (repetition time), 5 × 1 × 5 mm multivoxel, NSA 32. Spectra were fitted to a seven-term baseline to the selected peaks Cho (choline), Cr (creatine), and NAA (N-acetyl-aspartate) metabolites using the MR acquisition software (version R5.1). The metabolite concentration was normalized between subjects using the creatine concentration since it is relatively constant [65].

### 2.7. Locomotion Analysis

Locomotion analysis was conducted one week after injury and weekly until the eighth week by placing the animal on a plexiglass runway to record the inferior and lateral aspects of gait and in a plexiglass cylindrical arena to record open-field free movement [66]. The Basso Beattie Bresnahan (BBB) locomotor rating scale for rats was used to evaluate functional recovery [67]. The BBB scale consists of 22 points, where 0 corresponds to the total absence of movement and 21 to normal locomotion. Regarding the spinal cord transection model, critical levels of recovery are 4 (slight movement of the three joints of the hindlimb: hip, knee, and ankle), 7 (extensive movement of the three joints), and 9 (introduction of weight-supported steps). 

### 2.8. Histology, Immunofluorescence, and Confocal Microscopy

After the eight weeks of study, rats were anesthetized with 100 mg/kg of pentobarbital. Using a peristaltic pump at 18 mL/min, the rats were transcardially perfused with 250 mL of ice-cold 0.9% NaCl followed by 250 mL of ice-cold 4% PFA in PB. The spinal cord was extracted, and 1 cm rostral and 1 cm caudal to the injury was post-fixed in 4% PFA for 24 h at 4 °C. Samples were then transferred to 30% sucrose in PB and stored at 4 °C [68]. 

For immunofluorescence analysis, spinal cord sections were cut into 40-µm-thick longitudinal sections with a cryostat. Slices were blocked with 5% BSA in TBS with 0.5% (*v*/*v*) Triton X-100 and incubated with anti-MAP2 (1:200; Invitrogen, Waltham, MA, USA, PA5-17646) for 24 h. Slices were then washed three times with TBS followed by 2 h of incubation at room temperature with Alexa Fluor 546 anti-rabbit secondary antibody (1:300, ThermoFisher Scientific, Waltham, MA, USA, A11035) in TBS. Images were obtained using confocal microscopy (Zeiss LSM 800, Jena, Germany) [68]. An average of 32 optical slices were obtained every 1 μm for each Z-stack. 

Quantification and image analysis were performed with the image processing package Fiji for ImageJ 1.54f (NIH) and CellProfiler 4.2.5 software (Broad Institute, Inc., Cambridge, MA, USA). 

Z-stacks analysis was performed in AMIRA software (FEI, Houston, TX, USA, version 5.4.5) for immunofluorescence mark identification and 3D reconstruction. 

### 2.9. Statistical Analysis

GraphPad Prism 9.0.2 software was used to perform data analysis. Data normality was evaluated with the Shapiro–Wilk test. An analysis of variance (ANOVA) was used for parametric data analysis, followed by Tukey’s multiple comparisons post-hoc test, whereas the Kruskal–Wallis test was used for non-parametric data, followed by Dunn’s post hoc test for group comparisons. A two-way ANOVA was used to evaluate interaction effects in vivo by treatment and time factors. Differences were considered significant if *p* < 0.05. Data are presented as mean ± SEM. Significant differences are depicted as * *p* < 0.05, ** *p* < 0.01, *** *p* < 0.001, **** *p* < 0.0001.

## 3. Results

### 3.1. Scaffold Design and Rapid Tissue Response to Plasma-Modified Scaffolds

**Figure 1:**
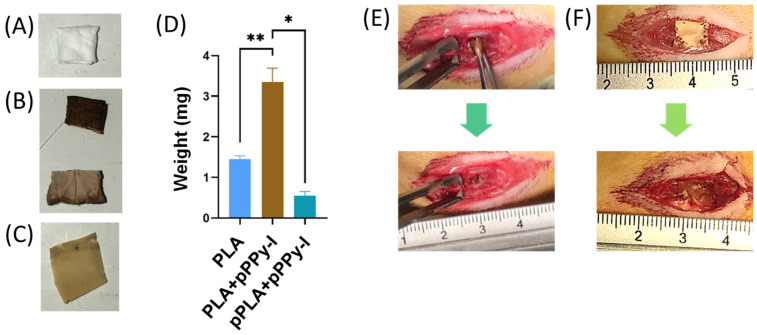
Representative images of the implants’ physical form: (**A**) PLA scaffold. (**B**) PLA + pPPy-I (top: exterior, bottom: inner layers). (**C**) PLA + pPPy-I patch (pPLA + pPPy-I). (**D**) Average weight (n = 4) of the constructs; * *p* < 0.05, ** *p* < 0.01. (**E**) Implant insertion between the spinal cord stumps. (**F**) Application of pPLA + pPPy-I. PLA (polylactic acid), PLA + pPPy-I (polylactic acid scaffold coated with plasma pyrrole polymer doped with iodine), pPLA + pPPy-I (a single layer of PLA scaffold coated with pPPy-I).

Engineered polylactic acid (PLA) scaffold implants consist of a four-layer randomly oriented fibers scaffold coated with a plasma pyrrole polymer doped with iodine (PLA + pPPy-I) (Figure 1A,B). The PLA + pPPy-I scaffolds present a more robust plasma polymer deposition at the exterior, while the inner layers are coated with a finer film (Figure 1B). Over 50% of the composite implant constitutes the plasma polymer (Figure 1D). A single layer of PLA was coated with pPPy-I (pPLA + pPPy-I), designed to function as a meninges repair patch (Figure 1C). The pPLA + pPPy-I was rapidly integrated into the meninges tissue, demonstrating the hydrophilicity of the pPPy-I (Figure 1E,F).

### 3.2. Implanted Animals Showed Improved Motor Recovery

**Figure 2:**
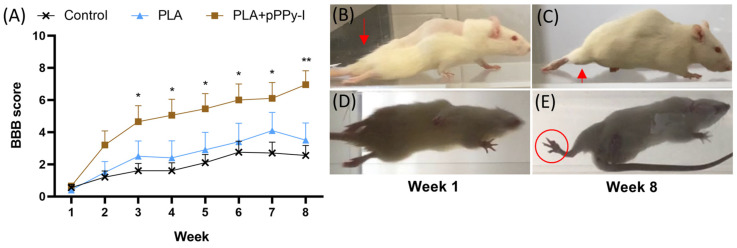
Functional motor recovery. (**A**) BBB locomotor scores throughout the eight weeks of study, data are presented as mean ± SEM, n = 10. A significant difference was found (* *p* < 0.05, ** *p* < 0.01) between Control and PLA + pPPy-I by the 2-way ANOVA test (week and treatment). Representative images of a PLA + pPPy-I-implanted rat one week after injury in (**B**,**D**), which moves to drag its hindlimbs without body weight support (red arrow); (**C**,**E**) the same rat in week 8, depicting plantar placement of the paw before raising the leg to take the step (red circle) with the support of body weight (red arrow). (**B**,**C**) Right-side view, (**D**,**E**) inferior view. SEM (standard error of the mean), ANOVA (analysis of variance), PLA (polylactic acid scaffold), PLA + pPPy-I (polylactic acid scaffold coated with plasma pyrrole polymer doped with iodine).

Despite the complete transection spinal cord injury, rats presented a functional motor response over time. Control animals presented a spontaneous response, which is common in this animal model [69]. PLA + pPPy-I-implanted animals presented a significantly superior functional response to control animals since week three of treatment (Figure 2, Video S2). Analysis within groups comparing the functional response over time showed no statistically significant differences in the Control or PLA groups. The performance of PLA-implanted and control animals remained similar from week 1 to week 8 in general (Video S1). In contrast, a significant difference was detected in the PLA + pPPy-I group from week 3 compared to week 1 (Video S1), suggesting that functional recovery was more efficient in the composite group, presenting significant functional results after three weeks of treatment.

A comparison with Intact animals after a spinal cord transection, one of the most severe models of SCI, was not performed as both sensory and motor tract continuity is eliminated [70].

### 3.3. Scaffolds Promoted Tissue Growth at the Injury Site

**Figure 3:**
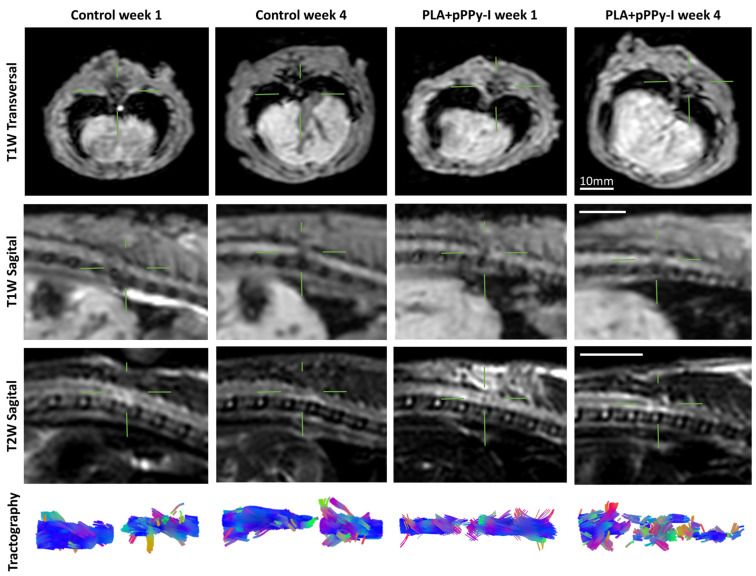
Representative images of Control and PLA + pPPy-I in the first and fourth weeks post-injury. PLA group images were similar to the composite group. Control animal showing hypointense T1W and hyperintense T2W signals at the injury site suggests hemorrhage or cerebrospinal fluid leak, which is dynamic over time. In the fourth week, the Control animal still presents pathological T1 hypointensity and T2 hyperintensity at the injury site. Hypointense T1 and T2 in the first week in the implanted animal depict the presence of the implant between the wholly transected spinal cord stumps. T1 and T2 intensity in the fourth week depicts tissue growth over the implant. Tractography confirms the information yielded by T1 and T2 images, increasing the resolution and showing tract microstructure. Bar = 10 mm. PLA + pPPy-I (polylactic acid scaffold coated with plasma pyrrole polymer doped with iodine), PLA (polylactic acid scaffold), T1W (T1-weighted), T1 (longitudinal relaxation time), T2W (T2-weighted), T2 (transverse relaxation time).

In the first week after injury, anatomical images showed T1 and T2 hypointensity contrast at the injury site in general, with some cases of T2 hyperintensity in the control group, which is associated with liquid tissue, such as cerebrospinal fluid leak, hemorrhage, edema, or cystic cavitation [12] (Figure 3). In the implanted animals, injury site hypointensity is associated with the presence of the implant, which emits no magnetic resonance (MR) signal. The evident gap between the spinal cord stumps confirmed the transection. By the fourth week after injury, PLA + pPPy-I-implanted animals showed T1 and T2 intensity associated with tissue covering the implant. In contrast, control and PLA-implanted animals showed evidence of secondary damage by T1 hypointensity colocalized with T2 hyperintensity, which sometimes extended to the spinal cord’s caudal portion.

Diffusion tensor imaging (DTI) enhances the sensitivity of magnetic resonance imaging (MRI), clearly showing the extent of spinal cord injury [71]. Tractography estimation of the injury site was performed using DTI data, which confirmed the complete transection of the spinal cord in week 1 of the study. In week 4, control and PLA-implanted animals still present a separation between spinal cord stumps. In contrast, PLA + pPPy-I animals present nerve tracts across stumps. 

### 3.4. Recovery of Structural Damage by the Fibrillar Scaffolds

**Figure 4:**
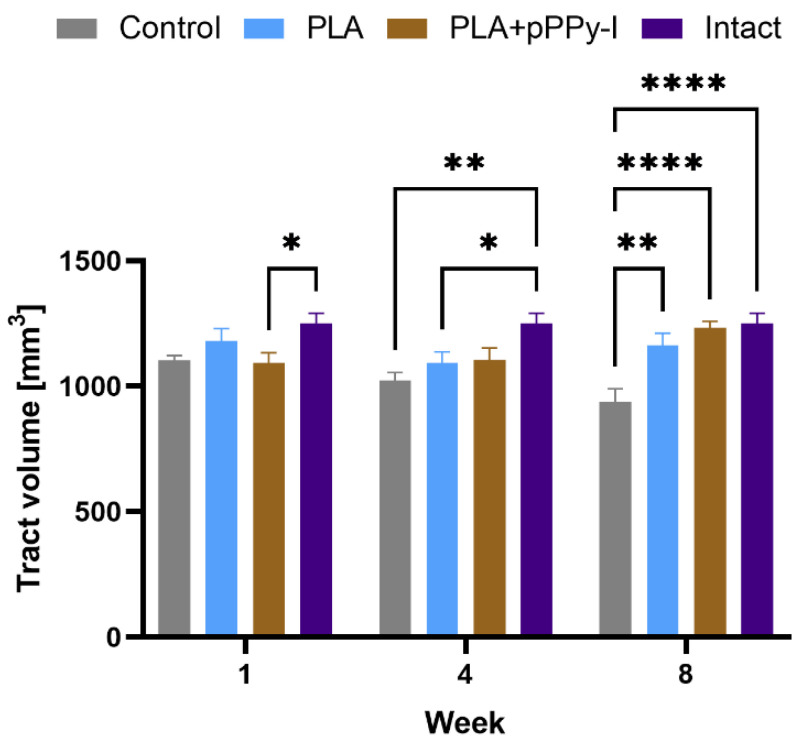
Tract volume (mm^3^) 1, 4, and 8 weeks post-injury. Statistical differences between groups by 2-way ANOVA, n = 5, * *p* < 0.05, ** *p* < 0.01, **** *p* < 0.0001. PLA + pPPy-I (polylactic acid scaffold coated with plasma pyrrole polymer doped with iodine), PLA (polylactic acid scaffold).

Using diffusion tensor imaging (DTI) data, we studied the impact of reconstructed tract volume over time between study groups (Figure 4). A significant decrease in tract volume in PLA + pPPy-I in week one is related to the complete transection and the lack of signal of the scaffold. In week 4, tract volume was recovered in PLA + pPPy-I, while a significant decrease in tract volume in control and PLA groups reflected the effects of secondary injury. In week 8, the tract volume of control animals decreased significantly concerning intact and scaffold-implanted groups. In contrast, implanted animals showed no statistical differences compared to the intact group, suggesting the implants provided a substrate for cell adhesion and neural tissue support.

Since tract volume significantly decreased in week 1 in PLA + pPPy-I, the recovered volume of tracts in the fourth week and a significant decrease in volume in PLA by week 4 suggest that the plasma-modified fibers promoted tissue growth through the scaffold, showing an improved performance compared to PLA scaffolds.

### 3.5. The Recovery of Anisotropy Baseline Values Suggests Neural Pathway Reorganization across the Scaffolds

**Figure 5:**
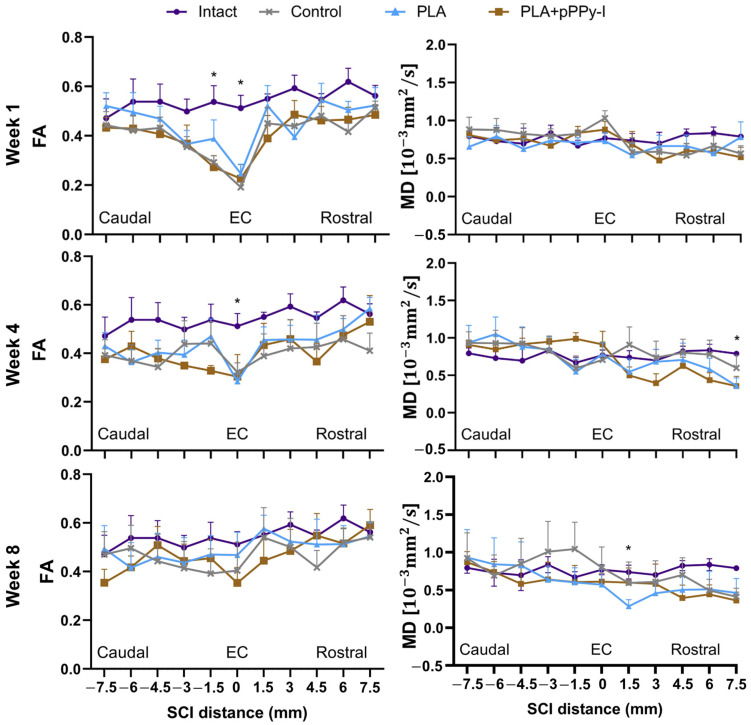
Diffusion tensor imaging indices FA (fractional anisotropy) and MD (mean diffusivity) at a 15 mm region of interest of the spinal cord, 0 = EC (the injury epicenter), 1, 4, and 8 weeks after injury. The 2-way ANOVA test found statistical differences, n = 5, * *p* < 0.05. FA (week 1): *p* = 0.049 PLA + pPPy-I vs. Intact 1.5mm caudal to EC; *p* = 0.0076 Control vs. Intact, *p* = 0.025 PLA + pPPy-I vs. Intact, *p* = 0.0167 Intact vs. PLA at EC. FA (week 4): *p* = 0.034 Intact vs. PLA at EC. MD (week 4): *p* = 0.045 Intact vs. PLA at the rostral side. MD (week 8): *p* = 0.029 1.5mm rostral to EC between Intact and PLA. PLA (polylactic acid scaffold), PLA + pPPy-I (polylactic acid scaffold coated with plasma pyrrole polymer doped with iodine).

The diffusion tensor imaging (DTI) information further complements magnetic resonance (MR) images since DTI yields water diffusion direction and DTI indices present quantitative measures of tissue microstructure. Diffusion indices were altered at the injury epicenter (EC) in week 1, depicting a general decrease in fractional anisotropy (FA). Mean diffusivity (MD) increased non-significantly in control and PLA + pPPy-I-implanted animals due to an increase in both axial and radial diffusivity (Figure 5). FA and MD values recovered by week 8, with the remaining MD significantly different to intact in PLA group 1.5 mm rostral to the injury epicenter, suggesting axonal damage in this region. 

The diffusion of water molecules yields indirect information about the tissue structure [71]. Fractional anisotropy (FA) increases linearly, such as in white matter tracts in the intact spinal cord. Due to spinal cord injury and physiopathologic processes, an FA decrease is expected. However, FA and mean diffusivity (MD) values may be modified. MD values remained similar to intact at the injury epicenter, suggesting water molecules remained restricted due to fibrotic scar formation in Control animals and tissue growth at the scaffolds in implanted animals. 

### 3.6. The Fibrillar Scaffolds Promoted Metabolic Changes in Response to Injury

**Figure 6:**
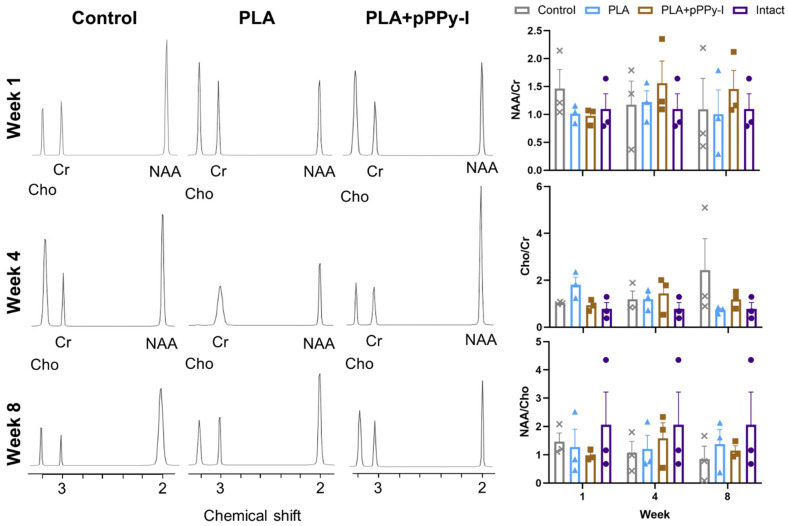
Representative spectra at the injury site and average ratios of NAA/Cr, Cho/Cr, and NAA/Cho of the Intact (•), Control (×), PLA-implanted (▲), PLA + pPPy-I-implanted (∎) animals 1, 4, and 8 weeks after injury (n = 3). N-acetyl-aspartate (NAA) appears at 2.01 ppm, Creatine (Cr) at 3.0 ppm, and Choline (Cho) at 3.2 ppm. Although no significant differences were found between groups, Cho/Cr ratio tended to increase in Control animals in week eight due to the secondary damage. PLA (polylactic acid scaffold), PLA + pPPy-I (polylactic acid scaffold coated with plasma pyrrole polymer doped with iodine).

Magnetic resonance (MR) spectra acquisition provides details of tissue constitution, increasing imaging sensitivity and conducing to more accurate diagnostics [47]. MR spectroscopy (MRS) provides information about molecular composition in tissue in vivo [72], which is especially valuable in longitudinal studies on the central nervous system as neural and axon integrity may be evaluated [73]. Metabolite concentrations were obtained from the MR spectra, and the relevant ratios of NAA/Cr, Cho/Cr, and NAA/Cho were calculated (Figure 6). 

Creatine (Cr) is a compound involved in energy metabolism. Cr is considered relatively constant and is therefore used as an internal concentration reference [65]. Although recent studies have found altered Cr levels in pathologic processes [72], it is used as a standard reference metabolite as growing evidence suggests metabolite/Cr ratios as potential markers of tissue physiopathology [47].

N-acetyl-aspartate (NAA) is a healthy neural tissue marker in healthy neurons, oligodendrocytes, and myelin. A decrease in NAA/Cr is associated with neuronal loss. NAA levels were still high one week after the injury. According to the literature, NAA molecules may remain trapped in neuronal debris in the acute phase and decrease in the subacute phase [73]. However, NAA values tend to normalize [73,74]. 

Choline (Cho) is a biomarker of cell membrane metabolism, reflecting cellular density and the rate of cellular membrane turnover. It is a marker in the synthesis and breakdown of cell membranes [73]. No significant differences were found between groups; however, Cho tends to increase in Control animals in week eight, which is expected due to secondary damage, suggesting demyelination in the chronic phase of SCI [73,74].

The NAA/Cho ratio has been proposed as a predictor of motor development in children by studying magnetic resonance (MR) spectra in the brain [75]. NAA/Cho has also been found to correlate to fractional anisotropy (FA). As NAA reflects healthy neural tissue and Cho is related to cell membrane metabolism, therefore the NAA/Cho ratio may suggest neural cell presence concerning gliosis as the inverse Cho/NAA has been associated with the extension of gliomas over healthy neural tissue [76,77]. Though not statistically significant, there was a marked decrease in NAA/Cho in week 1 in implanted animals, reflecting the signal decrease due to the implants. In week 4, NAA/Cho tended to increase in composite-implanted animals, likely due to an NAA increase and Cho decrease as a result of pPPy-I’s neuroprotective properties [78]. This plasma polymer might be acting as a free radical scavenger [79,80], moderating the secondary damage and reflecting a decrease in Cho. However, this hypothesis is currently under study. 

### 3.7. The Plasma Modification of the Fibers Mitigated Tissue Degeneration

**Figure 7:**
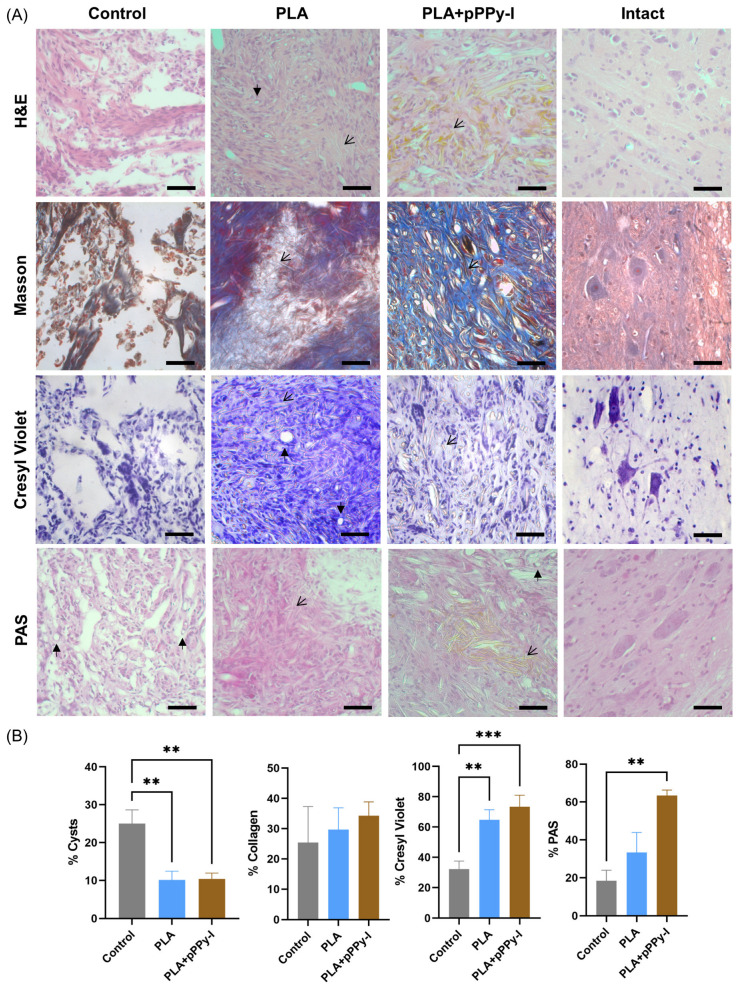
Representative histological images of the injury site in Control animals and PLA- and PLA + pPPy-I-implanted animals at the injury epicenter in (**A**), bar = 50 µm, 40× magnification, closed arrows: capillary, open arrows: fibers. Statistical analysis of histologic images at the epicenter and implants in (**B**). Percent of pathophysiologic biomarkers in the injury epicenter of 3 rats per group: cysts area (n = 9 frames per group), Cresyl violet (n = 9), and PAS-positive area (n = 3), ** *p* < 0.01, *** *p* < 0.001 by ANOVA and Tukey’s test; percent of the collagen-positive area (n = 6), without significant differences using Kruskal–Wallis and Dunn’s tests. PLA (polylactic acid scaffold), PLA + pPPy-I (polylactic acid scaffold coated with plasma pyrrole polymer doped with iodine), H&E (hematoxylin and eosin stain), PAS (Periodic acid–Schiff stain).

After eight weeks, spinal cord tissue was examined using different staining techniques to characterize the injury epicenter (Figure 7). Control images showed that transection only resulted in large cysts and deteriorated tissue in general. Significantly fewer cells and a significantly lower amount of basal lamina were found at the epicenter, as shown by the PAS-positive area (Figure 7B), compared to PLA + pPPy-I. Implants were covered by tissue in general, as depicted by H&E. However, control animals presented significantly higher cystic areas of degenerated tissue than scaffold-implanted animals. PLA implants showed large portions devoid of tissue, as shown by the PAS stain. However, all groups presented blood vessels at the epicenter and inside the implants. Masson’s trichrome stain showed that collagen type I was present in the scaffolds and the injury epicenter, likely due to fibroblast infiltration. PLA- and PLA + pPPy-I-implanted animals show significantly larger areas covered by neural tissue than the control, as depicted by the Cresyl Violet stain. 

**Figure 8:**
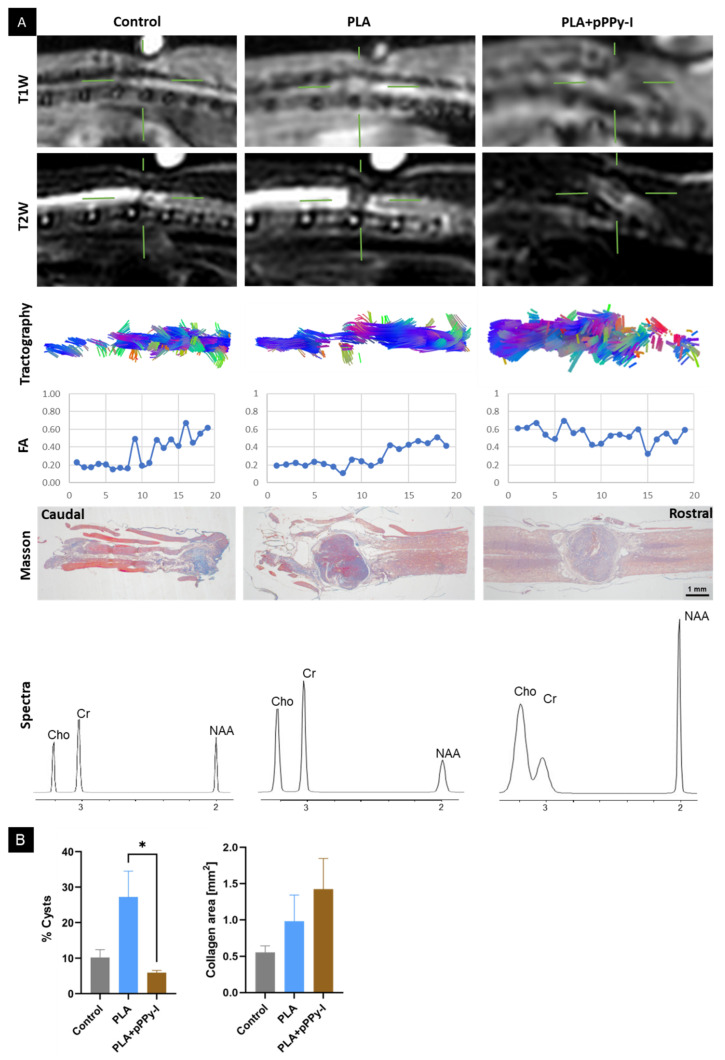
Representative images at eight weeks post-injury (**A**), comparing T1W, T2W, tractography, FA (of transversal regions from the caudal = 0 to the rostral = 20 side), Masson’s trichrome, and spectra of a control, PLA-, and PLA + pPPy-I-implanted rat. Green lines point to the injury epicenter. (**B**) Statistical analysis of whole histologic samples: percent of cysts (n = 3 frames/group), * *p* < 0.05 using Kruskal–Wallis and Dunn’s tests. Collagen area, n = 4 frames/group, no significant differences were found between groups by ANOVA. T1W (T1-weighted images), T2W (T2-weighted images), FA (fractional anisotropy), PLA (polylactic acid scaffold), PLA + pPPy-I (PLA coated with plasma pyrrole polymer doped with iodine).

Images of one animal from each group were compared eight weeks after injury (Figure 8). The techniques used in the present study reflect the structure of the spinal cord, further validating magnetic resonance (MR) studies from previous time points. As illustrated by the histologic sections, caudal tissue degeneration is extensive in control and PLA-implanted animals, depicted by large cysts and neural tissue deterioration, and thus DTI tractography presents a lack of tracts at the caudal side, as well as T2 hyperintensity and T1 hypointensity. In contrast, the PLA + pPPy-I-implanted animal shows smaller cysts surrounding the implant and T1 and T2 tissue-related intensity through the injury site and tract continuity, suggesting that the implant provided neuroprotection and prevented further tissue damage. Furthermore, the metabolite concentration was higher in the composite animal, which suggests a stronger signal of functional neural tissue in this case. 

Whereas differences in cyst percentage were significant between PLA and PLA + pPPy-I, no significant differences were found with the control, where cyst quantification was affected by tissue destruction in control samples, as depicted by histologic analysis.

### 3.8. Evidence of Neuronal Marker MAP2 over the Fibers Demonstrated Fibrillar Scaffolds Are Potential Substrates for Nerve Repair

**Figure 9:**
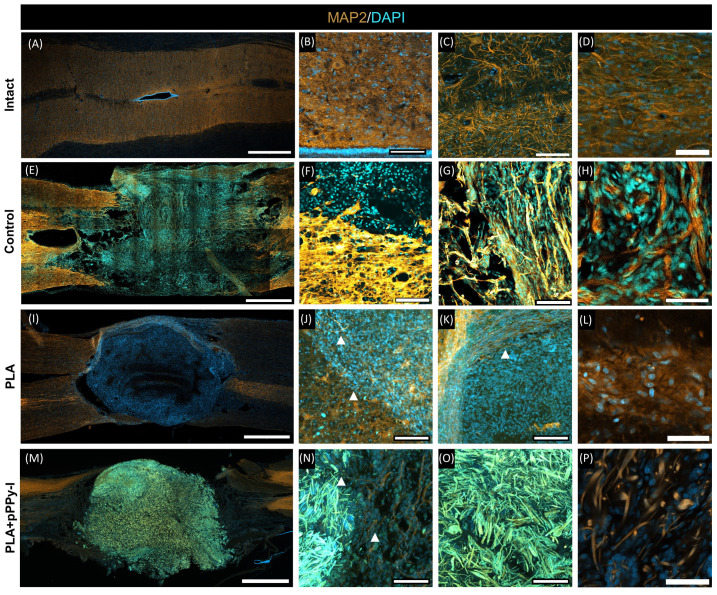
Representative images of MAP2/DAPI-labeled sections of the intact spinal cord, Control, PLA, and PLA + pPPy-I implants. (**A**,**E**,**I**,**M**) Bar = 1 mm. Close-up views of intact tissue in (**B**,**C**), control in (**F**,**G**), PLA implant in (**J**,**K**), PLA + pPPy-I implant in (**N**,**O**). (**B**,**C**,**F**,**G**,**J**,**K**,**N**,**O**) bar = 100 µm, 20× magnification, arrowheads = microtubules. Transition zone between spared and scar tissue in (**F**). Implant and adjacent tissue in (**J**,**N**). Detail of MAP2 mark in (**D**) intact, (**H**) Control, (**L**) PLA, (**P**) PLA + pPPy-I. (**D**,**H**,**L**,**P**) bar = 50 µm, 40× magnification. MAP2 (neuronal microtubules), DAPI (cells’ nuclei), PLA (polylactic acid scaffold), PLA + pPPy-I (PLA coated with plasma pyrrole polymer doped with iodine).

MAP2 is a neuronal marker in the dendrites of mature neurons [48], and DAPI is associated with the nucleus of cells. Figure 9 shows MAP2/DAPI expression in intact and PLA and PLA + pPPy-I-implanted animals. Intact spinal cord tissue depicts gray matter motoneurons and part of the ependymal canal, closely adjacent to the supporting glial cells (Figure 9A–D). MAP2 in intact spinal cords is expressed as a fiber mesh, whose density depends on its location, which is more densely expressed at the grey-matter region (Figure 9C). 

Upon spinal cord transection, a complete disruption of tissue organization and continuity was evidenced by the MAP2 mark (Figure 9E). As expected, some degree of neural regeneration is attempted from the rostral side of the injury [69], and disorganized microtubules extend over the fibrotic scar (Figure 9G, H), likely using the collagen fibers generated by fibroblast encroachment [81] as a substrate. However, large cysts were found on the caudal side of the injury (Figure 9E, left), forming a barrier and impeding neural tissue reconnection (Figure 9F).

The DAPI mark evidenced cell infiltration across the scaffolds. MAP2 expression inside and adjacent to the implants was also detected. In PLA samples, microtubules showed a disorganized structure over the randomly oriented fibers of the scaffold (Figure 9J, L). Adjacent to the scaffold, linearly organized MAP2 fibers were detected (Figure 9K). This mark suggests neurite extension was supported by the fibrillar scaffold over its periphery and through the scaffold. 

PLA + pPPy-I presents fluorescence (Figure 9I–K), which emits a signal in both DAPI and MAP2 channels. Thus, the immunofluorescence mark is not quantifiable in PLA + pPPy-I samples. However, MAP2/DAPI expression was evidenced across the implant over the coated fibers (Figure 9P).

**Figure 10:**
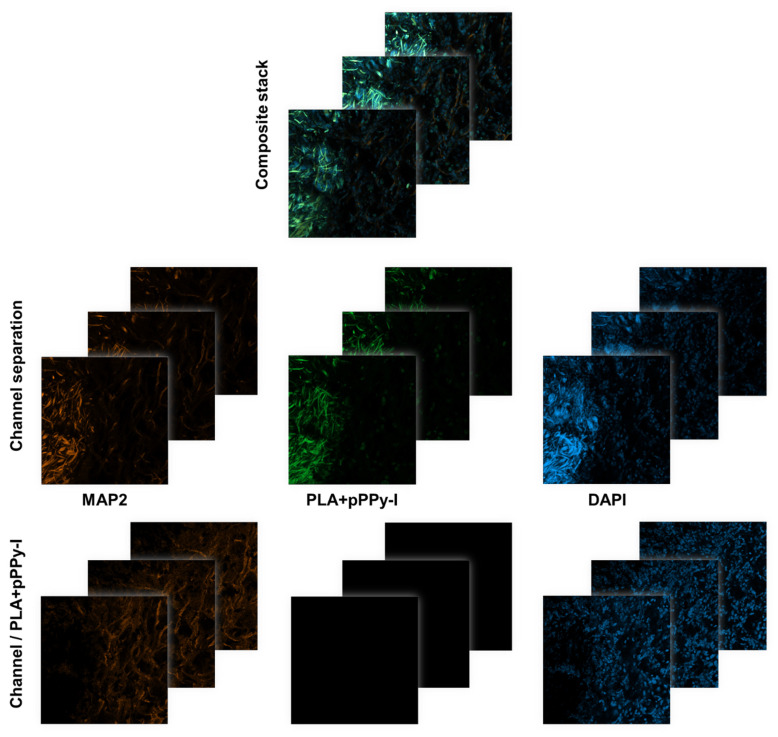
Immunofluorescence image processing. Confocal stacks (20× magnification) were separated by channels and the division operator was applied by optical slices. As a result, the PLA + pPPy-I fluorescence mark is removed from the images and the MAP2 and DAPI marks are identified. MAP2 (neuronal microtubules), DAPI (cells’ nuclei), PLA + pPPy-I (polylactic acid fibers coated with plasma pyrrole polymer doped with iodine).

To identify the immunofluorescence mark of MAP2 and DAPI, confocal stack images were separated by channels and a division operator was applied between optical slices to remove the pPPy-I fluorescence mark (Figure 10). 

**Figure 11:**
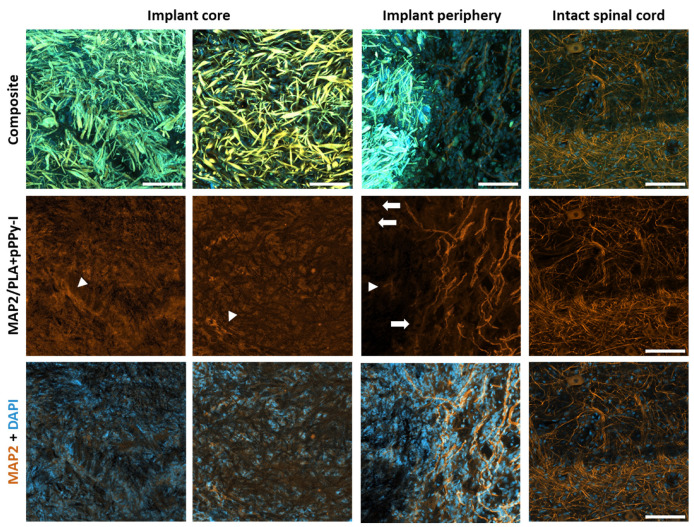
MAP2 and DAPI marks isolated from the pPPy-I fluorescence superposition. Representative images of the implant core and periphery regions showing neuronal microtubules and supporting cells. Intact region is presented for comparison of a neuron soma (block arrows) and neurite projections (arrowheads). Bar = 100 µm, 20× magnification. MAP2 (neuronal microtubules), DAPI (cells’ nuclei), pPPy-I (plasma pyrrole polymer doped with iodine).

Despite the potential loss of MAP2 and DAPI signals colocalized with the pPPy-I-coated fibers by this processing, it affords the actual presence of cells’ nuclei and neuronal microtubules adjacent to and within the PLA + pPPy-I implants, as shown in Figure 11.

**Figure 12:**
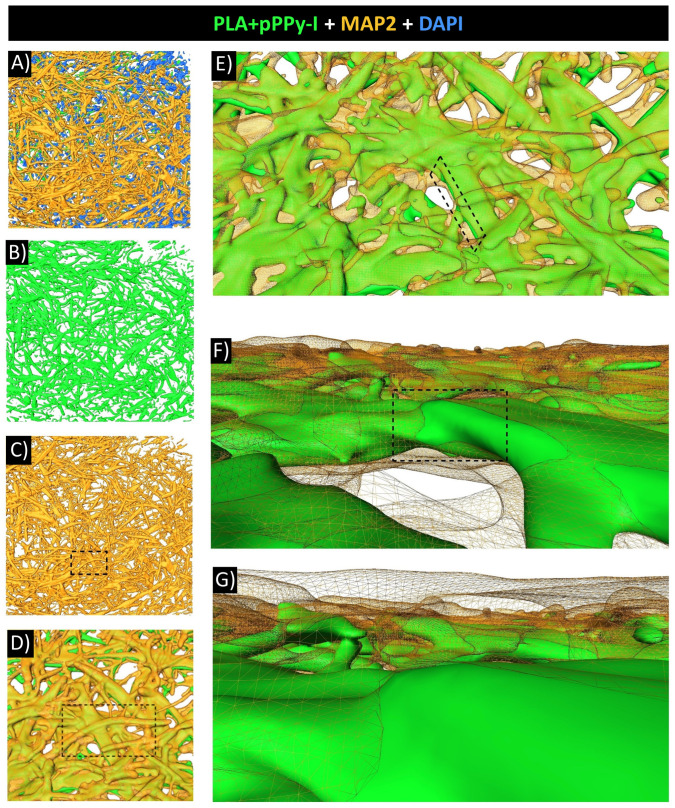
3D volumes of immunofluorescence MAP2 (neuronal microtubules) mark over the PLA + pPPy-I implant. (**A**) The implant core displays MAP2 and DAPI (nuclei) biomarkers over the PLA + pPPy-I fibers. (**B**) The PLA + pPPy-I portion is displayed. MAP2 volume portion is depicted in (**C**). Close-up views (dashed frames) of the same frame are shown in (**D**–**G**), focusing on the MAP2-PLA + pPPy-I interfaces. MAP2 (neuronal microtubules), DAPI (cells’ nuclei), PLA + pPPy-I (polylactic acid scaffold coated with plasma pyrrole polymer doped with iodine).

Three-dimensional (3D) analysis of confocal images was performed to reconstruct the region-of-interest volume. Optical slices 1 µm thick were separated by channels. The PLA + pPPy-I portion was identified using set theory as the signal present at the three channels by the intersection operation between the three sets of signals. Once the pPPy-I signal was identified and assigned to a 3D reconstructed volume, the MAP2 and DAPI independent signals were assigned as the sets of signals remaining once the pPPy-I mark was removed. Figure 12 shows reconstructed volumes of processed slides.

3D reconstruction of MAP2 mark over the PLA + pPPy-I fibers evidences neural tissue growth through the composite scaffolds and reaching the rostral and caudal sides of the implant. Using the image analysis pipeline proposed in this work, the MAP2 mark was identified from composite immunofluorescence images where the fluorescence of pPPy-I masks the neuronal-specific biomarker, which was found to cover the scaffold fibers. These results suggest that functional recovery was possible due to neurite extension over the PLA + pPPy-I scaffolds reconnecting neural spared tissue of the transected spinal cord.

## 4. Discussion

Spinal cord injury (SCI) constitutes a disruption in neural tissue integrity, cell death, axonal destruction, reactive astrocyte proliferation, failure of oligodendrocyte differentiation, and hence, demyelination, and results in autonomic and sensorimotor impairment [82,83]. Functional recovery from SCI, especially complete injuries, is currently an unmet medical problem. Approved therapies and clinical protocols have managed to reduce the incidence of complete SCI; however, efficient recovery for acute and chronic patients has not been accomplished [5]. 

Complete transection injuries are uncommon in humans; however, experimentally, they constitute a reliable model to investigate tissue regeneration. Complete transection models of spinal cord injury imply the complete disruption of neural tissue, thus preventing the transmission of any bioelectrical and biochemical signals, leading to motor and sensory loss. Incomplete injury models afford better prognosis since the remaining tracts promote circuit reorganization and favor sensory-motor recovery. Nevertheless, protecting spared tissue from the effects of secondary injury is crucial for plasticity processes to take place [20]. 

Spinal cord transection at the thoracic level produces paraplegia and sensory loss below the injury site. In the first week of the study, the animals had paralyzed hindlimbs, and motor recovery was observed over time. Rat SCI models are expected to present some level of spontaneous recovery [69], which was evidenced by the MAP2 mark over the fibrotic scar. Despite the MAP2 mark found at the injury epicenter in the control group, motor recovery was significantly impaired. 

Neural regeneration in the adult central nervous system is extremely difficult due to inhibitory signals [84], ineffective necrotic tissue debridement, chronic inflammation, and the formation of fluid-filled cavities that constitute a poor substrate for cell migration and neural tissue reconnection [1], which cause failure of regenerative impulses [85]. However, significant motor recovery was found in the PLA + pPPy-I group compared to the control group without treatment. After three weeks of treatment, the effects of the plasma polymer are reflected in significant functional motor recovery compared to the control without treatment, which is a relevant result since patients suffering from an SCI today have limited therapeutical options affording efficient results [20,24,86]. 

Fibrillar scaffolds provide a porous, permissive structure for tissue growth and have been proposed as a tissue engineering strategy for SCI in acute and chronic stages as they constitute a mechanically stable substrate for regeneration support [87]. However, the pursuit of an efficient composition and structure is a current research field. In the present work, we used a fibrillar, randomly aligned PLA scaffold coated with plasma pyrrole polymer doped with iodine, which has demonstrated neuroprotection and modulation of the secondary injury effects in complete transection and contusion SCI. Recently, gene expression promoted by the application of pPPy-I in SCI was characterized. Genes associated with neuron development, neurogenesis, neuronal differentiation, axonal growth, synapses, and synaptic vesicle transport were upregulated. They also found that pPPy-I promotes a decrease in apoptotic stimuli, which might shift the injury site microenvironment and promote neural growth factor expression [88].

The level of motor recovery achieved with the coated fibrillar scaffolds (PLA + pPPy-I) in complete transection injury models exceeds 3 points on average on the BBB locomotion rating scale compared to the pPPy-I tablet reported in previous studies [34,35]. This suggests that the fibrillar structure combined with the pPPy-I surface may enhance the functional effect due to the greater availability of pPPy-I to the cells, in conjunction with the structurally more stable substrate provided by the scaffold.

In the present study, we evaluated the performance of an implant without any biological components such as neural growth factors or cells or stimulating therapies (magnetic or electrical) and observed significant motor recovery compared to control animals. The design of the scaffold implant proposed affords versatility to these therapies to enhance the functional response, as the topography allows the addition or seeding of biological stimulators [7,32]. 

Magnetic resonance (MR) anatomical images revealed that by the fourth week of treatment, the PLA + pPPy-I implant was covered by tissue. The tractography analysis further verified this, where the projection of tracts through the implant site was evidenced. Diffusion tensor imaging information and tractography further complement MR images [89], affording structural organization by the water diffusion within the tissue. Despite no significant changes between weeks 4 and 8 by MR anatomical images, microstructural changes were evidenced by fractional anisotropy recovery. Additionally, while control animals presented a significant decrease in tract volume, implanted animals showed microstructural improvement, particularly in PLA + pPPy-I, suggesting sustained tissue growth at the implant, which may explain the significant functional recovery of the animals. 

In vivo analysis of pathophysiologic metabolites revealed NAA/Cr levels tend to recover, suggesting the presence of functional neural tissue [74,90]. Increased choline (Cho) is associated with cell membrane metabolism, gliosis, and demyelination. Since reactive astrocyte proliferation is part of the spinal cord response to injury in an attempt to control damage propagation, among other effects [91,92], increased levels of Cho are expected after an SCI. However, the Cho/Cr biomarker is expected to normalize. In this case, the control group shows a Cho/Cr tendency to increase, which is associated with demyelination at the chronic phase [73]. As NAA (N-acetyl aspartate) is the healthy neuronal tissue marker, the NAA/Cho ratio value reflects a balance of neurons and supporting glia, which is essential for nervous tissue functionality. Despite no statistical differences being found in this case, the MR protocol presented in this work aims for translational medicine applications since MR studies are among the most relevant in vivo diagnostics and prognostics tools, affording detailed tissue structure information [47]. 

Neural tissue reorganization is acutely activated after injury but persists over time. The implant provided a supporting substrate, as shown by the histology and immunofluorescence images. The implants were infiltrated by different types of cells and formed basal lamina, which likely promoted tissue adaptation and stimulated the regenerative capacity of the nervous tissue since the scaffolds provide a supportive matrix for glial cell adhesion and migration as well as neurite extension [93,94]. Further analysis using other techniques will be considered in future studies to demonstrate this hypothesis. 

The cystic cavities in control and PLA-implanted animals were significantly larger than in PLA + pPPy-I animals. Neuronal microtubules were evident in the PLA implant periphery, whereas the fluorescence in PLA + pPPy-I implants masked the immunofluorescent MAP2 mark. The image processing protocol presented in this work aimed to demonstrate the presence of neuronal microtubules since the number of fluorophores outside the pPPy-I fluorescence spectrum (350–600 nm) [95,96] is strikingly reduced. The fibrillar scaffold structure supported neurite extension between the spinal cord stumps, as shown by the MAP2 mark present over the fibers (Figure 11 and Figure 12, Video S3). Despite the fact that the MAP2 mark was also found in the injury epicenter of control animals, efficient functional recovery was not achieved. After three weeks of treatment, significant motor recovery was shown in the composite scaffold group. In vivo evaluation of the injury and recovery progression was achieved using MRI biomarkers, such as tract volume and DTI indices, which demonstrated that structural support was provided for functional neural tissue extension, as one of the possible recovery mechanisms. 

Free radicals are part of the hostile setting established by secondary damage, contributing to a pro-inflammatory environment in the injury epicenter, which collapses the nervous tissue’s attempts at recovery [1,22]. Conductive polymers such as polypyrrole and polyaniline have antioxidant properties due to the presence of amine (NH) groups in the polymer chains, which promotes the scavenging effect of free radicals, as well as slight changes in the polymer chains such as crosslinking [79,80]. Surface area is an important factor in the free radical scavenging effect. In the scaffolds presented in this work, the pyrrole polymer is available on a large surface area because it coats the surface of the fibers; hence, the effect in the tissue environment may be amplified [92]. A more detailed characterization of the scaffolds is reported elsewhere [44].

Substrate topography and electrical stimulation have been studied as cues for stem cell differentiation towards neuronal lineage, which express MAP2 and Tuj1 neuronal markers [48,97]. Since the spinal cord has stem cell niches [98,99], stimulation of inactive endogenous stem cells is a potential strategy for SCI recovery [100]. In this study, MAP2 expression was found over the fibers of the implant, suggesting neural extension through the scaffolds, which might contribute to spared tissue reconnection between the transected spinal cord stumps. As pPPy-I affords electroconductive properties in the physiological environment [50,101], bioelectrical cues might propagate through the scaffold coating, promoting the differentiation of endogenous stem cells. However, the elucidation of the effects of PLA + pPPy-I implants on endogenous stem cell differentiation and free radical scavenging properties is still under study, and the application of PCR or additional immunostaining analysis to demonstrate such recovery mechanisms will be considered in future work. Future work regarding such potential means of SCI recovery might further elucidate PLA + pPPy-I composite fibrillar scaffolds’ therapeutic efficacy.

Upon SCI, a large mass of neural tissue is lost due to necrosis, apoptosis, ischemia, chemical imbalance, and excitotoxicity. Among the most valuable clinical treatment strategies is surgical decompression within the first 24–48 h [5,17]. The primary injury evolves into the cascade of events of secondary injury, which culminates in the formation of a fibrotic scar, which constitutes a physical barrier that prevents regeneration attempts [1,2]. In this chronic scenario, surgical removal of this inhibitory tissue has been proposed [28]. In both the acute and the chronic phases of SCI, surgical removal of necrotic products has been proposed as a promising strategy when combined with the application of a scaffold with optimal properties to drive spinal cord recovery [18]. The results presented in this work show that PLA coated with pPPy-I affords rapid interaction with the host tissue, enhances hydrophilicity compared to PLA alone, promotes significant motor recovery compared to the control group receiving no implant, stimulates structural reorganization, constitutes a permissive substrate for neural tracts between the spinal stumps, and displays tissue volume comparable to intact animals, secondary injury moderation, and neural tissue infiltration to the scaffold.

## 5. Conclusions

Complete spinal cord injury produces a disconnection of neural tracts, hemorrhage, and inflammation and develops a secondary injury in control animals without treatment. The implantation of fibrillar scaffolds provided a substrate for cell attachment and cell infiltration through the pores of the scaffold. However, a significant reduction in cystic cavities was demonstrated by the pPPy-l coating while providing a permissive environment for neural tissue growth through the coated scaffold. In vivo analysis showed that improved microstructural recovery was facilitated after three weeks of treatment with the composite scaffold, accompanied by significant locomotor recovery.

## Data Availability

The datasets used and/or analyzed during the current study are available from the corresponding author upon reasonable request.

## Supplementary Materials

The following supporting information can be downloaded at: https://www.mdpi.com/article/10.3390/polym16081133/s1, Video S1: PLA + pPPy-I-implanted animal in week 1. Representative locomotion performance of the animals in week 1; regardless of the treatment, all the animals move dragging their hindlimbs without any bodyweight support from the thoracic spinal cord. Video S2: PLA + pPPy-I-implanted animal in week 8. In contrast to control animals, PLA + pPPy-I-implanted animals show significant motor recovery. Video S3: Representative confocal image processing showing fiber guidance on neural tissue extension across the PLA + pPPy-I implant. Magnetic resonance (MR) imaging analysis was performed in the experimental animals to assess the treatment response in vivo. Anatomical sequences are implemented to study the whole animal, whereas diffusion tensor imaging (DTI), MR Spectroscopy, and histologic and immunofluorescence analyses were focused on the injury epicenter. Immunofluorescence analysis demonstrated neuronal microtubules over the PLA + pPPy-I fibers, which, according to motor recovery, might explain the functional recovery mechanism of the fibrillar scaffold implant.

## References

[1] Ahuja C.S., Wilson J.R., Nori S., Kotter M.R.N., Druschel C., Curt A., Fehlings M.G. (2017). Traumatic Spinal Cord Injury. Nat. Rev. Dis. Prim..

[2] Alizadeh A., Dyck S.M., Karimi-Abdolrezaee S. (2019). Traumatic Spinal Cord Injury: An Overview of Pathophysiology, Models and Acute Injury Mechanisms. Front. Neurol..

[3] Bennett J., Das J., Emmady P. (2024). Spinal Cord Injuries. StatPearls [Internet].

[4] Flack J., Sharma K., Xie J. (2022). Delving into the Recent Advancements of Spinal Cord Injury Treatment: A Review of Recent Progress. Neural Regen. Res..

[5] Grijalva-Otero I., Doncel-Pérez E. (2024). Traumatic Human Spinal Cord Injury: Are Single Treatments Enough to Solve the Problem?. Arch. Med. Res..

[6] Zustiak S.P., Sheth S., Imaninezhad M. (2020). Pharmacological Therapies and Factors Delivery for Spinal Cord Injury Regeneration. Spinal Cord Injury (SCI) Repair Strategies.

[7] Hu X., Xu W., Ren Y., Wang Z., He X., Huang R., Ma B., Zhao J., Zhu R., Cheng L. (2023). Spinal Cord Injury: Molecular Mechanisms and Therapeutic Interventions. Signal Transduct. Target. Ther..

[8] Oña A., Athanasios K., Tederko P., Escorpizo R., Arora M., Sturm C., Yang S., Barzallo D.P. (2023). Unmet Healthcare Needs and Health Inequalities in People with Spinal Cord Injury: A Direct Regression Inequality Decomposition. Int. J. Equity Health.

[9] Forte G., Giuffrida V., Scuderi A., Pazzaglia M. (2022). Future Treatment of Neuropathic Pain in Spinal Cord Injury: The Challenges of Nanomedicine, Supplements or Opportunities?. Biomedicines.

[10] Guízar-Sahagún G., Grijalva I., Franco-Bourland R.E., Madrazo I. (2023). Aging with Spinal Cord Injury: A Narrative Review of Consequences and Challenges. Ageing Res. Rev..

[11] Patek M., Stewart M. (2023). Spinal Cord Injury. Anaesth. Intensive Care Med..

[12] Chandra J., Sheerin F., Lopez De Heredia L., Meagher T., King D., Belci M., Hughes R.J. (2012). MRI in Acute and Subacute Post-Traumatic Spinal Cord Injury: Pictorial Review. Spinal Cord.

[13] Kim K.D., Lee K.S., Coric D., Harrop J.S., Theodore N., Toselli R.M. (2022). Acute Implantation of a Bioresorbable Polymer Scaffold in Patients with Complete Thoracic Spinal Cord Injury: 24-Month Follow-up from the INSPIRE Study. Neurosurgery.

[14] Morales-Guadarrama A., Salgado-Ceballos H., Grijalva I., Morales-Corona J., Hernández-Godínez B., Ibáñez-Contreras A., Ríos C., Diaz-Ruiz A., Cruz G.J., Olayo M.G. (2022). Evolution of Spinal Cord Transection of Rhesus Monkey Implanted with Polymer Synthesized by Plasma Evaluated by Diffusion Tensor Imaging. Polymers.

[15] Theodore N., Hlubek R., Danielson J., Neff K., Vaickus L., Ulich T.R., Ropper A.E. (2016). First Human Implantation of a Bioresorbable Polymer Scaffold for Acute Traumatic Spinal Cord Injury: A Clinical Pilot Study for Safety and Feasibility. Neurosurgery.

[16] Guest J.D., Moore S.W., Aimetti A.A., Kutikov A.B., Santamaria A.J., Hofstetter C.P., Ropper A.E., Theodore N., Ulich T.R., Layer R.T. (2018). Internal Decompression of the Acutely Contused Spinal Cord: Differential Effects of Irrigation Only versus Biodegradable Scaffold Implantation. Biomaterials.

[17] Kumar R., Lim J., Mekary R.A., Rattani A., Dewan M.C., Sharif S.Y., Osorio-Fonseca E., Park K.B. (2018). Traumatic Spinal Injury: Global Epidemiology and Worldwide Volume. World Neurosurg..

[18] Wang N., Xiao Z., Zhao Y., Wang B., Li X., Li J., Dai J. (2018). Collagen Scaffold Combined with Human Umbilical Cord-Derived Mesenchymal Stem Cells Promote Functional Recovery after Scar Resection in Rats with Chronic Spinal Cord Injury. J. Tissue Eng. Regen. Med..

[19] Zhao Y., Tang F., Xiao Z., Han G., Wang N., Yin N., Chen B., Jiang X., Yun C., Han W. (2017). Clinical Study of NeuroRegen Scaffold Combined with Human Mesenchymal Stem Cells for the Repair of Chronic Complete Spinal Cord Injury. Cell Transplant..

[20] Shen H., Fan C., You Z., Xiao Z., Zhao Y., Dai J. (2022). Advances in Biomaterial-Based Spinal Cord Injury Repair. Adv. Funct. Mater..

[21] Gelain F., Panseri S., Antonini S., Cunha C., Donega M., Lowery J., Taraballi F., Cerri G., Montagna M., Baldissera F. (2011). Transplantation of Nanostructured Composite Scaffolds Results in the Regeneration of Chronically Injured Spinal Cords. ACS Nano.

[22] Mutepfa A.R., Hardy J.G., Adams C.F. (2022). Electroactive Scaffolds to Improve Neural Stem Cell Therapy for Spinal Cord Injury. Front. Med. Technol..

[23] Cheng Y., Zhang Y., Wu H. (2022). Polymeric Fibers as Scaffolds for Spinal Cord Injury: A Systematic Review. Front. Bioeng. Biotechnol..

[24] Suzuki H., Imajo Y., Funaba M., Ikeda H., Nishida N., Sakai T. (2023). Current Concepts of Biomaterial Scaffolds and Regenerative Therapy for Spinal Cord Injury. Int. J. Mol. Sci..

[25] Kaplan B., Levenberg S. (2022). The Role of Biomaterials in Peripheral Nerve and Spinal Cord Injury: A Review. Int. J. Mol. Sci..

[26] Faccendini A., Vigani B., Rossi S., Sandri G., Bonferoni M.C., Caramella C.M., Ferrari F. (2017). Nanofiber Scaffolds as Drug Delivery Systems to Bridge Spinal Cord Injury. Pharmaceuticals.

[27] Kim K.D., Lee K.S., Coric D., Chang J.J., Harrop J.S., Theodore N., Toselli R.M. (2021). A Study of Probable Benefit of a Bioresorbable Polymer Scaffold for Safety and Neurological Recovery in Patients with Complete Thoracic Spinal Cord Injury: 6-Month Results from the INSPIRE Study. J. Neurosurg. Spine.

[28] Xiao Z., Tang F., Tang J., Yang H., Zhao Y., Chen B., Han S., Wang N., Li X., Cheng S. (2016). One-Year Clinical Study of NeuroRegen Scaffold Implantation Following Scar Resection in Complete Chronic Spinal Cord Injury Patients. Sci. China Life Sci..

[29] Sánchez-Torres S., Díaz-Ruíz A., Ríos C., Olayo M.G., Cruz G.J., Olayo R., Morales J., Mondragón-Lozano R., Fabela-Sánchez O., Orozco-Barrios C. (2020). Recovery of Motor Function after Traumatic Spinal Cord Injury by Using Plasma-Synthesized Polypyrrole/Iodine Application in Combination with a Mixed Rehabilitation Scheme. J. Mater. Sci. Mater. Med..

[30] Álvarez-Mejía L., Morales J., Cruz G.J., Olayo M.-G., Olayo R., Díaz-Ruíz A., Ríos C., Mondragón-Lozano R., Sánchez-Torres S., Morales-Guadarrama A. (2015). Functional Recovery in Spinal Cord Injured Rats Using Polypyrrole/Iodine Implants and Treadmill Training. J. Mater. Sci. Mater. Med..

[31] Mondragon-Lozano R., Ríos C., Roldan-Valadez E., Cruz G.J., Olayo M.G., Olayo R., Salgado-Ceballos H., Morales J., Mendez-Armenta M., Alvarez-Mejia L. (2017). Delayed Injection of Polypyrrole Doped with Iodine Particle Suspension after Spinal Cord Injury in Rats Improves Functional Recovery and Decreased Tissue Damage Evaluated by 3.0 Tesla in Vivo Magnetic Resonance Imaging. Spine J..

[32] Fabela-Sánchez O., Salgado–Ceballos H., Medina-Torres L., Álvarez-Mejía L., Sánchez-Torres S., Mondragón-Lozano R., Morales-Guadarrama A., Díaz-Ruiz A., Olayo M.-G., Cruz G.J. (2018). Effect of the Combined Treatment of Albumin with Plasma Synthesised Pyrrole Polymers on Motor Recovery after Traumatic Spinal Cord Injury in Rats. J. Mater. Sci. Mater. Med..

[33] Olayo R., Ríos C., Salgado-Ceballos H., Cruz G.J., Morales J., Olayo M.G., Alcaraz-Zubeldia M., Alvarez A.L., Mondragon R., Morales A. (2008). Tissue Spinal Cord Response in Rats after Implants of Polypyrrole and Polyethylene Glycol Obtained by Plasma. J. Mater. Sci. Mater. Med..

[34] Cruz G.J., Mondragón-Lozano R., Diaz-Ruiz A., Manjarrez J., Olayo R., Salgado-Ceballos H., Olayo M.G., Morales J., Alvarez-Mejía L., Morales A. (2012). Plasma Polypyrrole Implants Recover Motor Function in Rats after Spinal Cord Transection. J. Mater. Sci. Mater. Med..

[35] Álvarez-Mejía L., Salgado-Ceballos H., Olayo R., Cruz G.J., Olayo M.G., Díaz-Ruiz A., Ríos C., Mondragón-Lozano R., Morales-Guadarrama A., Sánchez-Torres S. (2015). Effect of Pyrrole Implants Synthesized by Different Methods on Spinal Cord Injuries of Rats. Rev. Mex. Ing. Biomed..

[36] Buzoianu-Anguiano V., Rivera-Osorio J., Orozco-Suárez S., Vega-García A., García-Vences E., Sánchez-Torres S., Jiménez-Estrada I., Guizar-Sahagún G., Mondragon-Caso J., Fernández-Valverde F. (2020). Single vs. Combined Therapeutic Approaches in Rats With Chronic Spinal Cord Injury. Front. Neurol..

[37] Hurtado A., Cregg J.M., Wang H.B., Wendell D.F., Oudega M., Gilbert R.J., McDonald J.W. (2011). Robust CNS Regeneration after Complete Spinal Cord Transection Using Aligned Poly-l-Lactic Acid Microfibers. Biomaterials.

[38] Cai J., Ziemba K.S., Smith G.M., Jin Y. (2007). Evaluation of Cellular Organization and Axonal Regeneration through Linear PLA Foam Implants in Acute and Chronic Spinal Cord Injury. J. Biomed. Mater. Res. Part A.

[39] Saini P., Arora M., Kumar M.N.V.R. (2016). Poly(Lactic Acid) Blends in Biomedical Applications. Adv. Drug Deliv. Rev..

[40] Ramezani Dana H., Ebrahimi F. (2023). Synthesis, Properties, and Applications of Polylactic Acid-Based Polymers. Polym. Eng. Sci..

[41] Ahmad A., Banat F., Alsafar H., Hasan S.W. (2024). An Overview of Biodegradable Poly (Lactic Acid) Production from Fermentative Lactic Acid for Biomedical and Bioplastic Applications. Biomass Convers. Biorefin..

[42] Pattanayak I., Alex Y., Mohanty S. (2023). Advancing Strategies towards the Development of Tissue Engineering Scaffolds: A Review. J. Mater. Sci..

[43] Hanumantharao S.N., Rao S. (2019). Multi-Functional Electrospun Nanofibers from Polymer Blends for Scaffold Tissue Engineering. Fibers.

[44] Osorio-Londoño D.M., Godínez-Fernández J.R., Acosta-García M.C., Morales-Corona J., Olayo-González R., Morales-Guadarrama A. (2021). Pyrrole Plasma Polymer-Coated Electrospun Scaffolds for Neural Tissue Engineering. Polymers.

[45] Younes H.M., Kadavil H., Ismail H.M., Adib S.A., Zamani S., Alany R.G., Al-Kinani A.A. (2024). Overview of Tissue Engineering and Drug Delivery Applications of Reactive Electrospinning and Crosslinking Techniques of Polymeric Nanofibers with Highlights on Their Biocompatibility Testing and Regulatory Aspects. Pharmaceutics.

[46] Alves C.M., Yang Y., Marton D., Carnes D.L., Ong J.L., Sylvia V.L., Dean D.D., Reis R.L., Agrawal C.M. (2008). Plasma Surface Modification of Poly(D,L-Lactic Acid) as a Tool to Enhance Protein Adsorption and the Attachment of Different Cell Types. J. Biomed. Mater. Res.-Part B Appl. Biomater..

[47] Martin A.R., Aleksanderek I., Cohen-Adad J., Tarmohamed Z., Tetreault L., Smith N., Cadotte D.W., Crawley A., Ginsberg H., Mikulis D.J. (2016). Translating State-of-the-Art Spinal Cord MRI Techniques to Clinical Use: A Systematic Review of Clinical Studies Utilizing DTI, MT, MWF, MRS, and FMRI. NeuroImage Clin..

[48] Garrudo F.F.F., Mikael P.E., Rodrigues C.A.V., Udangawa R.W., Paradiso P., Chapman C.A., Hoffman P., Colaço R., Cabral J.M.S., Morgado J. (2021). Polyaniline-Polycaprolactone Fibers for Neural Applications: Electroconductivity Enhanced by Pseudo-Doping. Mater. Sci. Eng. C.

[49] Osorio-Londono D.M., Sanchez-Morales G.S., Garcia-Garcia G., Morales-Guadarrama A., Olayo-Gonzalez R. Pyrrole Plasma Polymer-Coated Fibrillar Scaffold Implant: Pilot Study in Rat Spinal Cord Transection with MRI. Proceedings of the 2021 43rd Annual International Conference of the IEEE Engineering in Medicine & Biology Society (EMBC).

[50] Cruz G.J., Morales J., Olayo R. (1999). Films Obtained by Plasma Polymerization of Pyrrole. Thin Solid Films.

[51] Serratos I.N., Olayo R., Millán-Pacheco C., Morales-Corona J., Vicente-Escobar J.O., Soto-Estrada A.M., Córdoba-Herrera J.G., Uribe O., Gómez-Quintero T., Arroyo-Ornelas M.Á. (2019). Modeling Integrin and Plasma-Polymerized Pyrrole Interactions: Chemical Diversity Relevance for Cell Regeneration. Sci. Rep..

[52] Van der Meulen A. (2014). The Effects of Switching Light-Dark Regime on the Behavior of Wistar Rats. Master’s Thesis.

[53] Roedel A., Storch C., Holsboer F., Ohl F. (2006). Effects of Light or Dark Phase Testing on Behavioural and Cognitive Performance in DBA Mice. Lab. Anim..

[54] Bertoglio L.J., Carobrez A.P. (2002). Behavioral Profile of Rats Submitted to Session 1-Session 2 in the Elevated plus-Maze during Diurnal/Nocturnal Phases and under Different Illumination Conditions. Behav. Brain Res..

[55] Committee for the Update of the Guide for the Care and Use of Laboratory Animals (2011). Guide for the Care and Use of Laboratory Animals.

[56] Kjell J., Olson L. (2016). Rat Models of Spinal Cord Injury: From Pathology to Potential Therapies. Dis. Model. Mech..

[57] Kosenko P.O., Smolikov A.B., Voynov V.B., Shaposhnikov P.D., Saevskiy A.I., Kiroy V.N. (2020). Effect of Xylazine–Tiletamine–Zolazepam on the Local Field Potential of the Rat Olfactory Bulb. Comp. Med..

[58] Sykes M., Matheson N.A., Brownjohn P.W., Tang A.D., Rodger J., Shemmeii J.B.H., Reynolds J.N.J. (2016). Differences in Motor Evoked Potentials Induced in Rats by Transcranial Magnetic Stimulation under Two Separate Anesthetics: Implications for Plasticity Studies. Front. Neural Circuits.

[59] Lukovic D., Moreno-Manzano V., Lopez-Mocholi E., Rodriguez-Jiménez F.J., Jendelova P., Sykova E., Oria M., Stojkovic M., Erceg S. (2015). Complete Rat Spinal Cord Transection as a Faithful Model of Spinal Cord Injury for Translational Cell Transplantation. Sci. Rep..

[60] Ramsey J.B.G., Ramer L.M., Inskip J.A., Alan N., Ramer M.S., Krassioukov A.V. (2010). Care of Rats with Complete High-Thoracic Spinal Cord Injury. J. Neurotrauma.

[61] Sotocinal S.G., Sorge R.E., Zaloum A., Tuttle A.H., Martin L.J., Wieskopf J.S., Mapplebeck J.C.S., Wei P., Zhan S., Zhang S. (2011). The Rat Grimace Scale: A Partially Automated Method for Quantifying Pain in the Laboratory Rat via Facial Expressions. Mol. Pain.

[62] Talbot S.R., Biernot S., Bleich A., van Dijk R.M., Ernst L., Häger C., Helgers S.O.A., Koegel B., Koska I., Kuhla A. (2020). Defining Body-Weight Reduction as a Humane Endpoint: A Critical Appraisal. Lab. Anim..

[63] Yeh F.-C., Verstynen T.D., Wang Y., Fernández-Miranda J.C., Tseng W.-Y.I. (2013). Deterministic Diffusion Fiber Tracking Improved by Quantitative Anisotropy. PLoS ONE.

[64] Yeh F.C., Panesar S., Barrios J., Fernandes D., Abhinav K., Meola A., Fernandez-Miranda J.C. (2019). Automatic Removal of False Connections in Diffusion MRI Tractography Using Topology-Informed Pruning (TIP). Neurotherapeutics.

[65] de Graaf R.A. (2007). In Vivo NMR Spectroscopy: Principles and Techniques.

[66] Brown A.R., Martinez M. (2019). Thoracic Spinal Cord Hemisection Surgery and Open-Field Locomotor Assessment in the Rat. J. Vis. Exp..

[67] Basso M.D., Beattie M.S., Bresnahan J.C. (1995). A Sensitive and Reliable Locomotor Rating Scale for Open Field Testing in Rats. J. Neurotrauma.

[68] Heras-Romero Y., Morales-Guadarrama A., Santana-Martínez R., Ponce I., Rincón-Heredia R., Poot-Hernández A.C., Martínez-Moreno A., Urrieta E., Bernal-Vicente B.N., Campero-Romero A.N. (2021). Improved Post-Stroke Spontaneous Recovery by Astrocytic Extracellular Vesicles. Mol. Ther..

[69] Han Q., Jin W., Xiao Z., Ni H., Wang J., Kong J., Wu J., Liang W., Chen L., Zhao Y. (2010). The Promotion of Neural Regeneration in an Extreme Rat Spinal Cord Injury Model Using a Collagen Scaffold Containing a Collagen Binding Neuroprotective Protein and an EGFR Neutralizing Antibody. Biomaterials.

[70] Antri M., Orsal D., Barthe J.Y. (2002). Locomotor Recovery in the Chronic Spinal Rat: Effects of Long-Term Treatment with a 5-HT2 Agonist. Eur. J. Neurosci..

[71] Hendrix P., Griessenauer C.J., Cohen-Adad J., Rajasekaran S., Cauley K.A., Shoja M.M., Pezeshk P., Tubbs R.S. (2015). Spinal Diffusion Tensor Imaging: A Comprehensive Review with Emphasis on Spinal Cord Anatomy and Clinical Applications. Clin. Anat..

[72] Cecil K.M. (2013). Proton Magnetic Resonance Spectroscopy: Technique for the Neuroradiologist. Neuroimaging Clin. N. Am..

[73] Qian J., Herrera J.J., Narayana P.A. (2010). Neuronal and Axonal Degeneration in Experimental Spinal Cord Injury: In Vivo Proton Magnetic Resonance Spectroscopy and Histology. J. Neurotrauma.

[74] Erschbamer M., Öberg J., Westman E., Sitnikov R., Olson L., Spenger C. (2011). 1H-MRS in Spinal Cord Injury: Acute and Chronic Metabolite Alterations in Rat Brain and Lumbar Spinal Cord. Eur. J. Neurosci..

[75] Kendall G.S., Melbourne A., Johnson S., Price D., Bainbridge A., Gunny R., Huertas-Ceballos A., Cady E.B., Ourselin S., Marlow N. (2014). White Matter NAA/Cho and Cho/Cr Ratios at MR Spectroscopy Are Predictive of Motor Outcome in Preterm Infants. Radiology.

[76] Cui Y., Zeng W., Jiang H., Ren X., Lin S., Fan Y., Liu Y., Zhao J. (2020). Higher Cho/NAA Ratio in Postoperative Peritumoral Edema Zone Is Associated With Earlier Recurrence of Glioblastoma. Front. Neurol..

[77] Guo J., Yao C., Chen H., Zhuang D., Tang W., Ren G., Wang Y., Wu J., Huang F., Zhou L. (2012). The Relationship between Cho/NAA and Glioma Metabolism: Implementation for Margin Delineation of Cerebral Gliomas. Acta Neurochir..

[78] Abbas W.A., Ibrahim M.E., El-Naggar M., Abass W.A., Abdullah I.H., Awad B.I., Allam N.K. (2020). Recent Advances in the Regenerative Approaches for Traumatic Spinal Cord Injury: Materials Perspective. ACS Biomater. Sci. Eng..

[79] Zare E.N., Agarwal T., Zarepour A., Pinelli F., Zarrabi A., Rossi F., Ashrafizadeh M., Maleki A., Shahbazi M.A., Maiti T.K. (2021). Electroconductive Multi-Functional Polypyrrole Composites for Biomedical Applications. Appl. Mater. Today.

[80] Hsu C.F., Peng H., Basle C., Travas-Sejdic J., Kilmartin P.A. (2011). ABTS•+ Scavenging Activity of Polypyrrole, Polyaniline and Poly(3,4-Ethylenedioxythiophene). Polym. Int..

[81] De Winter F., Oudega M., Lankhorst A.J., Hamers F.P., Blits B., Ruitenberg M.J., Pasterkamp R.J., Gispen W.H., Verhaagen J. (2002). Injury-Induced Class 3 Semaphorin Expression in the Rat Spinal Cord. Exp. Neurol..

[82] Papa S., Mauri E., Rossi F., Perale G., Veglianese P. (2020). Introduction to Spinal Cord Injury as Clinical Pathology. Spinal Cord Injury (SCI) Repair Strategies.

[83] Hou S., Rabchevsky A.G. (2014). Autonomic Consequences of Spinal Cord Injury. Compr. Physiol..

[84] Yiu G., He Z. (2006). Glial Inhibition of CNS Axon Regeneration. Nat. Rev. Neurosci..

[85] Harel N.Y., Strittmatter S.M. (2006). Can Regenerating Axons Recapitulate Developmental Guidance during Recovery from Spinal Cord Injury?. Nat. Rev. Neurosci..

[86] Rowald A., Komi S., Demesmaeker R., Baaklini E., Hernandez-Charpak S.D., Paoles E., Montanaro H., Cassara A., Becce F., Lloyd B. (2022). Activity-Dependent Spinal Cord Neuromodulation Rapidly Restores Trunk and Leg Motor Functions after Complete Paralysis. Nat. Med..

[87] Yu H., Yang S., Li H., Wu R., Lai B., Zheng Q. (2023). Activating Endogenous Neurogenesis for Spinal Cord Injury Repair: Recent Advances and Future Prospects. Neurospine.

[88] Coyoy-Salgado A., Orozco-Barrios C., Sánchez-Torres S., Olayo M.G., Cruz G.J., Morales-Corona J., Olayo R., Diaz-Ruiz A., Ríos C., Alvarez-Mejia L. (2023). Gene Expression and Locomotor Recovery in Adult Rats with Spinal Cord Injury and Plasma-Synthesized Polypyrrole/Iodine Application Combined with a Mixed Rehabilitation Scheme. Front. Neurol..

[89] Vedantam A., Jirjis M.B., Schmit B.D., Wang M.C., Ulmer J.L., Kurpad S.N. (2014). Diffusion Tensor Imaging of the Spinal Cord: Insights From Animal and Human Studies. Neurosurgery.

[90] Moffett J., Ross B., Arun P., Madhavarao C., Namboodiri A. (2007). N-Acetylaspartate in the CNS: From Neurodiagnostics to Neurobiology. Prog. Neurobiol..

[91] Yang T., Dai Y.J., Chen G., Cui S. (2020). Sen Dissecting the Dual Role of the Glial Scar and Scar-Forming Astrocytes in Spinal Cord Injury. Front. Cell. Neurosci..

[92] Yu G.L., Zhang Y., Ning B. (2021). Reactive Astrocytes in Central Nervous System Injury: Subgroup and Potential Therapy. Front. Cell. Neurosci..

[93] Vipin A., Thow X.Y., Mir H., Kortelainen J., Manivannan J., Al-Nashash H., All A.H. (2016). Natural Progression of Spinal Cord Transection Injury and Reorganization of Neural Pathways. J. Neurotrauma.

[94] Aguilar J., Humanes-Valera D., Alonso-Calviño E., Yague J.G., Moxon K.A., Oliviero A., Foffani G. (2010). Spinal Cord Injury Immediately Changes the State of the Brain. J. Neurosci..

[95] Vasquez-Ortega M., Ortega M., Morales J., Olayo M.G., Cruz G.J., Olayo R. (2014). Core-Shell Polypyrrole Nanoparticles Obtained by Atmospheric Pressure Plasma Polymerization. Polym. Int..

[96] Yang P., Zhang J., Guo Y. (2009). Synthesis of Intrinsic Fluorescent Polypyrrole Nanoparticles by Atmospheric Pressure Plasma Polymerization. Appl. Surf. Sci..

[97] Du B.L., Zeng C.G., Zhang W., Quan D.P., Ling E.A., Zeng Y.S. (2014). A Comparative Study of Gelatin Sponge Scaffolds and PLGA Scaffolds Transplanted to Completely Transected Spinal Cord of Rat. J. Biomed. Mater. Res.-Part A.

[98] Li X., Liu D., Xiao Z., Zhao Y., Han S., Chen B., Dai J. (2019). Scaffold-Facilitated Locomotor Improvement Post Complete Spinal Cord Injury: Motor Axon Regeneration versus Endogenous Neuronal Relay Formation. Biomaterials.

[99] Wang Y., Yuan H. (2022). Research Progress of Endogenous Neural Stem Cells in Spinal Cord Injury. Ibrain.

[100] Fan C., Li X., Xiao Z., Zhao Y., Liang H., Wang B., Han S., Li X., Xu B., Wang N. (2017). A Modified Collagen Scaffold Facilitates Endogenous Neurogenesis for Acute Spinal Cord Injury Repair. Acta Biomater..

[101] Colín E., Olayo M.G., Cruz G.J., Carapia L., Morales J., Olayo R. (2009). Affinity of Amine-Functionalized Plasma Polymers with Ionic Solutions Similar to Those in the Human Body. Prog. Org. Coat..

